# Supplementary material for: Modeling glioblastoma heterogeneity as a dynamic network of cell states
Source: Mol Syst Biol. 2021 Sep 16;17(9):e10105. doi: 10.15252/msb.202010105 (PMC8444284; doi:10.15252/msb.202010105)
Supplement: Supplementary file 5 — Source Data for Figure 3 [file MSB-17-e10105-s001.zip › Figure3A_sourcedata/GSEA_3065/hallmarks_state5.GseaPreranked.1623416302988/HALLMARK_DNA_REPAIR.html]

Details for gene set HALLMARK\_DNA\_REPAIR[GSEA]

|  || Dataset | state5 |
| Phenotype | NoPhenotypeAvailable |
| Upregulated in class | na\_neg |
| GeneSet | HALLMARK\_DNA\_REPAIR |
| Enrichment Score (ES) | -0.52010757 |
| Normalized Enrichment Score (NES) | -1.8398174 |
| Nominal p-value | 0.0 |
| FDR q-value | 1.875E-4 |
| FWER p-Value | 0.001 |
Table: GSEA Results Summary

  

Fig 1: Enrichment plot: HALLMARK\_DNA\_REPAIR      
 Profile of the Running ES Score & Positions of GeneSet Members on the Rank Ordered List

  

| PROBE | GENE SYMBOL | GENE\_TITLE | RANK IN GENE LIST | RANK METRIC SCORE | RUNNING ES | CORE ENRICHMENT || 1 | DGCR8 |  |  | 194 | 0.442 | -0.0053 | No |
| 2 | ARL6IP1 |  |  | 232 | 0.418 | 0.0061 | No |
| 3 | BCAP31 |  |  | 235 | 0.415 | 0.0214 | No |
| 4 | SEC61A1 |  |  | 406 | 0.338 | 0.0149 | No |
| 5 | DAD1 |  |  | 721 | 0.257 | -0.0110 | No |
| 6 | ADCY6 |  |  | 782 | 0.245 | -0.0086 | No |
| 7 | DDB2 |  |  | 833 | 0.235 | -0.0054 | No |
| 8 | PDE4B |  |  | 968 | 0.215 | -0.0125 | No |
| 9 | NELFCD |  |  | 969 | 0.215 | -0.0045 | No |
| 10 | REV3L |  |  | 1140 | 0.193 | -0.0165 | No |
| 11 | RNMT |  |  | 1163 | 0.190 | -0.0118 | No |
| 12 | NUDT9 |  |  | 1213 | 0.183 | -0.0105 | No |
| 13 | POM121 |  |  | 1285 | 0.173 | -0.0120 | No |
| 14 | NT5C3A |  |  | 1535 | 0.148 | -0.0346 | No |
| 15 | SRSF6 |  |  | 1558 | 0.146 | -0.0316 | No |
| 16 | NT5C |  |  | 1569 | 0.144 | -0.0273 | No |
| 17 | BCAM |  |  | 1575 | 0.143 | -0.0225 | No |
| 18 | NME3 |  |  | 1877 | 0.120 | -0.0520 | No |
| 19 | GTF3C5 |  |  | 2222 | 0.097 | -0.0872 | No |
| 20 | CANT1 |  |  | 2299 | 0.092 | -0.0924 | No |
| 21 | TMED2 |  |  | 2321 | 0.091 | -0.0914 | No |
| 22 | TK2 |  |  | 2356 | 0.088 | -0.0919 | No |
| 23 | CSTF3 |  |  | 2411 | 0.084 | -0.0948 | No |
| 24 | VPS37B |  |  | 2904 | 0.058 | -0.1482 | No |
| 25 | POLL |  |  | 2982 | 0.053 | -0.1549 | No |
| 26 | ERCC4 |  |  | 3047 | 0.050 | -0.1603 | No |
| 27 | LIG1 |  |  | 3181 | 0.042 | -0.1737 | No |
| 28 | SNAPC4 |  |  | 3201 | 0.041 | -0.1743 | No |
| 29 | RFC4 |  |  | 3212 | 0.041 | -0.1739 | No |
| 30 | TAF1C |  |  | 3257 | 0.038 | -0.1775 | No |
| 31 | NFX1 |  |  | 3425 | 0.031 | -0.1952 | No |
| 32 | NCBP2 |  |  | 3487 | 0.028 | -0.2010 | No |
| 33 | UPF3B |  |  | 3527 | 0.026 | -0.2045 | No |
| 34 | ERCC2 |  |  | 3674 | 0.021 | -0.2202 | No |
| 35 | PRIM1 |  |  | 3702 | 0.020 | -0.2225 | No |
| 36 | POLR1C |  |  | 3793 | 0.016 | -0.2320 | No |
| 37 | RFC3 |  |  | 3863 | 0.013 | -0.2393 | No |
| 38 | UMPS |  |  | 4211 | -0.002 | -0.2784 | No |
| 39 | POLD1 |  |  | 4333 | -0.007 | -0.2918 | No |
| 40 | XPC |  |  | 4377 | -0.009 | -0.2963 | No |
| 41 | AK3 |  |  | 4543 | -0.016 | -0.3144 | No |
| 42 | GTF2H1 |  |  | 4600 | -0.018 | -0.3200 | No |
| 43 | AAAS |  |  | 4706 | -0.022 | -0.3310 | No |
| 44 | GSDME |  |  | 4779 | -0.025 | -0.3382 | No |
| 45 | ERCC3 |  |  | 4795 | -0.025 | -0.3390 | No |
| 46 | POLA1 |  |  | 4853 | -0.028 | -0.3444 | No |
| 47 | SF3A3 |  |  | 4875 | -0.028 | -0.3457 | No |
| 48 | ERCC8 |  |  | 5075 | -0.036 | -0.3668 | No |
| 49 | DDB1 |  |  | 5186 | -0.040 | -0.3777 | No |
| 50 | ELL |  |  | 5210 | -0.041 | -0.3788 | No |
| 51 | POLA2 |  |  | 5229 | -0.042 | -0.3793 | No |
| 52 | TP53 |  |  | 5275 | -0.043 | -0.3828 | No |
| 53 | NUDT21 |  |  | 5349 | -0.047 | -0.3893 | No |
| 54 | POLD3 |  |  | 5393 | -0.048 | -0.3923 | No |
| 55 | GMPR2 |  |  | 5513 | -0.053 | -0.4037 | No |
| 56 | DCTN4 |  |  | 5560 | -0.055 | -0.4069 | No |
| 57 | TARBP2 |  |  | 5627 | -0.059 | -0.4121 | No |
| 58 | POLR2A |  |  | 5681 | -0.061 | -0.4158 | No |
| 59 | POLB |  |  | 5975 | -0.073 | -0.4461 | No |
| 60 | ELOA |  |  | 6040 | -0.076 | -0.4505 | No |
| 61 | POLR2D |  |  | 6062 | -0.077 | -0.4500 | No |
| 62 | RFC5 |  |  | 6186 | -0.084 | -0.4607 | No |
| 63 | GTF2H3 |  |  | 6221 | -0.086 | -0.4613 | No |
| 64 | RRM2B |  |  | 6229 | -0.086 | -0.4589 | No |
| 65 | SMAD5 |  |  | 6355 | -0.093 | -0.4695 | No |
| 66 | SUPT5H |  |  | 6427 | -0.097 | -0.4739 | No |
| 67 | ZWINT |  |  | 6453 | -0.099 | -0.4730 | No |
| 68 | POLR2C |  |  | 6493 | -0.101 | -0.4736 | No |
| 69 | TAF13 |  |  | 6544 | -0.105 | -0.4754 | No |
| 70 | RAE1 |  |  | 6601 | -0.108 | -0.4776 | No |
| 71 | RPA2 |  |  | 6705 | -0.114 | -0.4850 | No |
| 72 | POLR2G |  |  | 6813 | -0.122 | -0.4925 | No |
| 73 | GTF2B |  |  | 6903 | -0.129 | -0.4977 | No |
| 74 | POLR2H |  |  | 7048 | -0.142 | -0.5087 | No |
| 75 | ITPA |  |  | 7128 | -0.148 | -0.5121 | Yes |
| 76 | USP11 |  |  | 7153 | -0.151 | -0.5091 | Yes |
| 77 | FEN1 |  |  | 7201 | -0.155 | -0.5086 | Yes |
| 78 | SDCBP |  |  | 7222 | -0.158 | -0.5050 | Yes |
| 79 | NELFB |  |  | 7334 | -0.172 | -0.5111 | Yes |
| 80 | TAF6 |  |  | 7401 | -0.181 | -0.5117 | Yes |
| 81 | SNAPC5 |  |  | 7465 | -0.188 | -0.5118 | Yes |
| 82 | POLR1D |  |  | 7494 | -0.190 | -0.5079 | Yes |
| 83 | GTF2F1 |  |  | 7581 | -0.201 | -0.5101 | Yes |
| 84 | VPS28 |  |  | 7650 | -0.211 | -0.5098 | Yes |
| 85 | ERCC1 |  |  | 7735 | -0.223 | -0.5110 | Yes |
| 86 | TAF9 |  |  | 7817 | -0.238 | -0.5112 | Yes |
| 87 | POLR3GL |  |  | 7822 | -0.238 | -0.5027 | Yes |
| 88 | SAC3D1 |  |  | 7825 | -0.240 | -0.4940 | Yes |
| 89 | NELFE |  |  | 7882 | -0.251 | -0.4909 | Yes |
| 90 | TAF12 |  |  | 7897 | -0.255 | -0.4830 | Yes |
| 91 | MPG |  |  | 7900 | -0.256 | -0.4736 | Yes |
| 92 | EIF1B |  |  | 7931 | -0.262 | -0.4672 | Yes |
| 93 | SURF1 |  |  | 7943 | -0.264 | -0.4586 | Yes |
| 94 | IMPDH2 |  |  | 7948 | -0.266 | -0.4491 | Yes |
| 95 | CETN2 |  |  | 7970 | -0.270 | -0.4414 | Yes |
| 96 | HPRT1 |  |  | 7974 | -0.271 | -0.4316 | Yes |
| 97 | TSG101 |  |  | 7995 | -0.275 | -0.4236 | Yes |
| 98 | SUPT4H1 |  |  | 8026 | -0.281 | -0.4164 | Yes |
| 99 | RFC2 |  |  | 8049 | -0.285 | -0.4083 | Yes |
| 100 | PCNA |  |  | 8082 | -0.293 | -0.4009 | Yes |
| 101 | DUT |  |  | 8118 | -0.300 | -0.3936 | Yes |
| 102 | SSRP1 |  |  | 8178 | -0.320 | -0.3883 | Yes |
| 103 | MRPL40 |  |  | 8236 | -0.335 | -0.3822 | Yes |
| 104 | GTF2H5 |  |  | 8345 | -0.368 | -0.3806 | Yes |
| 105 | GTF2A2 |  |  | 8390 | -0.385 | -0.3712 | Yes |
| 106 | APRT |  |  | 8406 | -0.390 | -0.3583 | Yes |
| 107 | MPC2 |  |  | 8433 | -0.401 | -0.3462 | Yes |
| 108 | POLR2K |  |  | 8439 | -0.403 | -0.3317 | Yes |
| 109 | DGUOK |  |  | 8451 | -0.408 | -0.3177 | Yes |
| 110 | RALA |  |  | 8460 | -0.414 | -0.3031 | Yes |
| 111 | POLR2E |  |  | 8500 | -0.434 | -0.2913 | Yes |
| 112 | COX17 |  |  | 8525 | -0.446 | -0.2773 | Yes |
| 113 | ALYREF |  |  | 8535 | -0.449 | -0.2615 | Yes |
| 114 | POLE4 |  |  | 8536 | -0.451 | -0.2447 | Yes |
| 115 | ADRM1 |  |  | 8548 | -0.458 | -0.2288 | Yes |
| 116 | RPA3 |  |  | 8572 | -0.471 | -0.2137 | Yes |
| 117 | NME1 |  |  | 8573 | -0.471 | -0.1961 | Yes |
| 118 | TYMS |  |  | 8599 | -0.488 | -0.1807 | Yes |
| 119 | POLR2F |  |  | 8607 | -0.496 | -0.1629 | Yes |
| 120 | POLR2I |  |  | 8641 | -0.519 | -0.1472 | Yes |
| 121 | TAF10 |  |  | 8775 | -0.630 | -0.1386 | Yes |
| 122 | RBX1 |  |  | 8776 | -0.631 | -0.1150 | Yes |
| 123 | EDF1 |  |  | 8789 | -0.643 | -0.0923 | Yes |
| 124 | POLR2J |  |  | 8798 | -0.648 | -0.0689 | Yes |
| 125 | NME4 |  |  | 8824 | -0.678 | -0.0464 | Yes |
| 126 | GUK1 |  |  | 8897 | -0.770 | -0.0257 | Yes |
| 127 | GPX4 |  |  | 8966 | -0.946 | 0.0020 | Yes |
Table: GSEA details [plain text format]

  

Fig 2: HALLMARK\_DNA\_REPAIR: Random ES distribution      
 Gene set null distribution of ES for **HALLMARK\_DNA\_REPAIR**
